# Mucus plug-induced obstruction in acute appendicitis

**DOI:** 10.1055/a-2433-1332

**Published:** 2024-10-25

**Authors:** Zijian Qiu, Fan Wang, Qiu Zhao, Hongling Wang

**Affiliations:** 1 89674 Department of Gastroenterology, Zhongnan Hospital of Wuhan University, Wuhan, China; 2 Hubei Clinical Center and Key Lab of Intestinal and Colorectal Diseases, Wuhan, China

A 53-year-old man was admitted for mid-upper abdominal pain and constipation for three days. The patient had a history of imperforate anus surgery. After admission, barium enema examination indicated localized stenosis of the anal canal and difficult defecation of contrast material. Colonoscopy showed the postoperative annus and appendiceal orifice edema with purulent secretion. With the possibility of appendicitis considered, endoscopic retrograde appendicitis therapy (ERAT) using an appendoscope (eyeMAX, 9-French; Micro-Tech (Nanjing) Co., Ltd., China) was prepared subsequently 1 .

During the operation, the appendoscope was inserted into the appendiceal lumen and a long strip of vitrina from the appendix opening to the terminus was detected ( Video 1 , Fig. 1 , Fig. 2 ). The vitrina was flushed out by the lavage of 0.5% metronidazole. In addition to mucous hyperemia, no neoplasm was found in the following appendix exploration ( Fig. 3 ). The patient’s abdominal pain was relieved immediately after the procedure. Finally, the vitrina was pathologically confirmed to be denatured necrosis ( Fig. 4 ). The patient was discharged three days later, and no recurrence or any other adverse event was noted during a two-month follow-up. To the best of our knowledge, this is the first reported endoscopic diagnosis and treatment of mucus plug-induced obstruction in acute appendicitis.

Endoscopic diagnosis and treatment of mucus plug-induced obstruction in acute appendicitis.Video 1

**Fig. 1:**
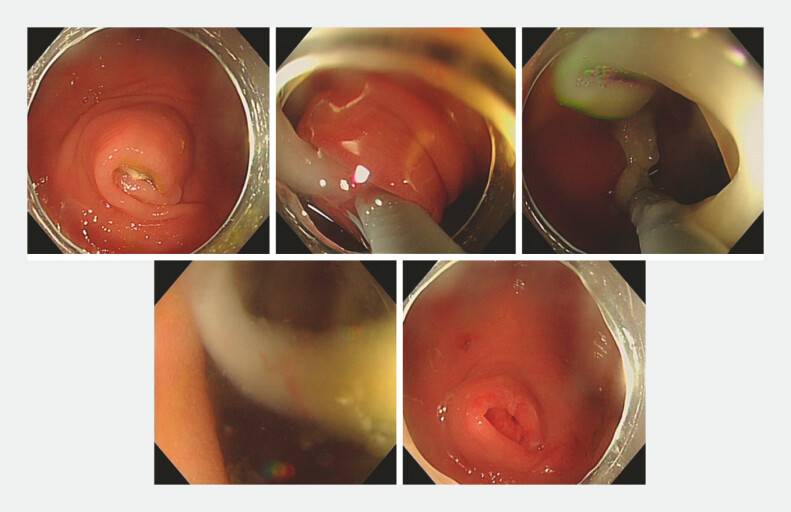
Appendoscope passing through the appendiceal orifice and flushing out a long strip of vitrina.

**Fig. 2:**
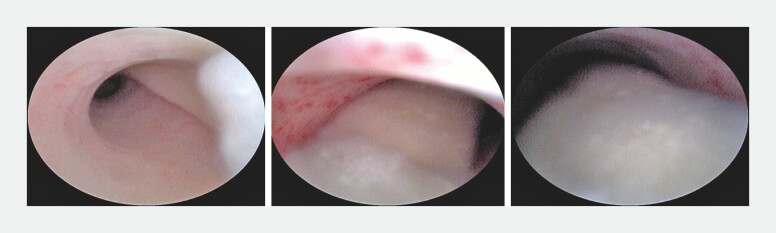
Detecting a long strip of vitrina in the appendiceal lumen.

**Fig. 3:**
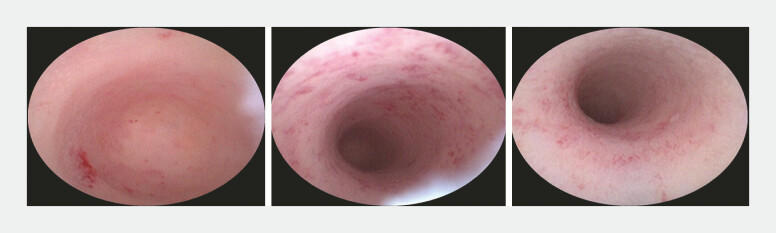
Exploring the appendix after removing the vitrina.

**Fig. 4:**
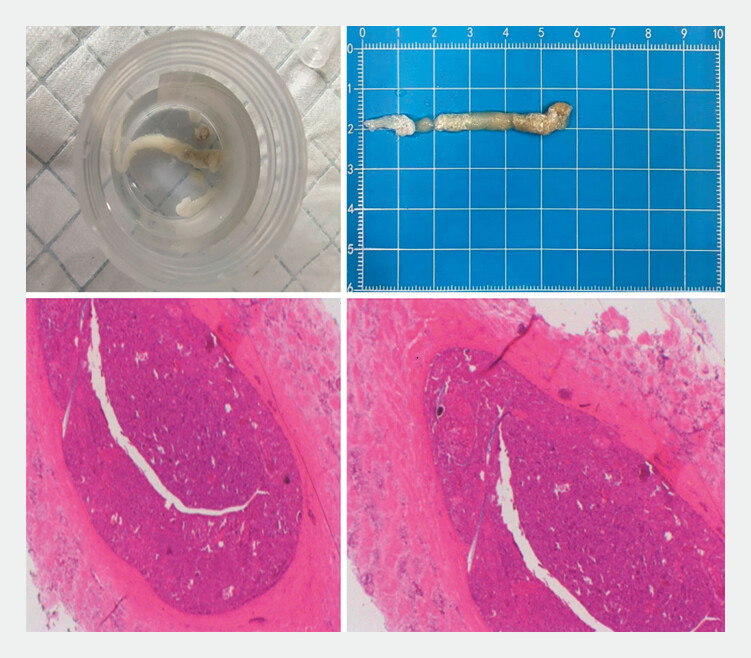
Pathological examination of the vitrina.

Endoscopy_UCTN_Code_TTT_1AQ_2AF

## Notes

Endoscopy E-Videos https://eref.thieme.de/e-videos
*E-Videos* is an open access online section of the journal *Endoscopy* , reporting on interesting cases and new techniques in gastroenterological endoscopy. All papers include a high-quality video and are published with a Creative Commons CC-BY license. Endoscopy E-Videos qualify for HINARI discounts and waivers and eligibility is automatically checked during the submission process. We grant 100% waivers to articles whose corresponding authors are based in Group A countries and 50% waivers to those who are based in Group B countries as classified by Research4Life (see: https://www.research4life.org/access/eligibility/ ). This section has its own submission website at https://mc.manuscriptcentral.com/e-videos .

## References

[1] Wang F Zhu Y Zhao Q Multifocal stenosis in purulent appendicitis with fecalith Endoscopy 2024 56 E108 E109 38307112 10.1055/a-2239-3401 PMC10837025

